# Ligneous periodontitis: A molecularly confirmed case of type I plasminogen deficiency 

**DOI:** 10.4317/jced.62773

**Published:** 2025-06-01

**Authors:** Atanur Sarioglu, Kubra Ugurlu, Meltem Karaman, Taner Karakaya, Tugrul Kirtiloglu, Ayse Zeynep Zengin

**Affiliations:** 1 Department of Periodontology, Faculty of Dentistry, Ondokuz Mayis University, Samsun, Turkey; 2 Department of Oral and Maxillofacial Surgery, Faculty of Dentistry, Ondokuz Mayis University, Samsun, Turkey; 3 Department of Oral and Maxillofacial Radiology, Faculty of Dentistry, Ondokuz Mayis University, Samsun, Turkey; 4 Department of Medical Genetics, Samsun Education and Research Hospital, Samsun, Turkey

## Abstract

Congenital plasminogen (PLG) deficiency is an exceptionally uncommon hereditary disease associated with biallelic pathogenic / likely pathogenic variants in the PLG gene. Ligneous periodontitis (LP) is a rare disorder that can occur as a result of a lack of plasminogen. It is defined by the presence of lobulated, membranous, and ulcerated masses in the gums, as well as significant damage to the surrounding bone. This case report presents the diagnosis, treatment, and follow-up outcomes of a 23-year-old male patient who referred to our clinic with a complaint of widespread gingival enlargement. We identified a novel c.2087G>C (p.Arg696Pro) variant with a known variant detected in a compound heterozygous state in PLG confirming the molecular etiology. This case report emphasizes the importance of dentists identifying oral manifestations of various systemic diseases. Careful examination of such findings and the timely referral of patients to appropriate specialists for diagnosis and treatment are of critical significance.

** Key words:**Ligneous periodontitis, plasminogen deficiency, genetic confirmation, histopathological diagnosis.

## Introduction

Type I PLG deficiency is a rare autosomal recessive disorder characterized by chronic pseudomembranous lesions in various mucosal sites, including the oral cavity, conjunctiva, and respiratory and genital tracts (1). The oral manifestations of type I PLG deficiency were first reported in detail by Gunhan *et al*., who introduced the term LP to describe a destructive membranous periodontal disease characterized by nodular gingival overgrowths and progressive alveolar bone destruction due to amyloid-like fibrin accumulation (2). In the 2022 systematic review by Alberto *et al*., focusing on case reports and series of ligneous gingival disorders, it is noTable that 53.1% of the reported cases originated from the Turkish population (3). The aim of this case report is to highlight the clinical presentation, diagnosis, multidisciplinary management, and long-term follow-up of a patient with LP, confirmed by molecular analysis of the PLG gene.

## Case Report

**Figure 1:**
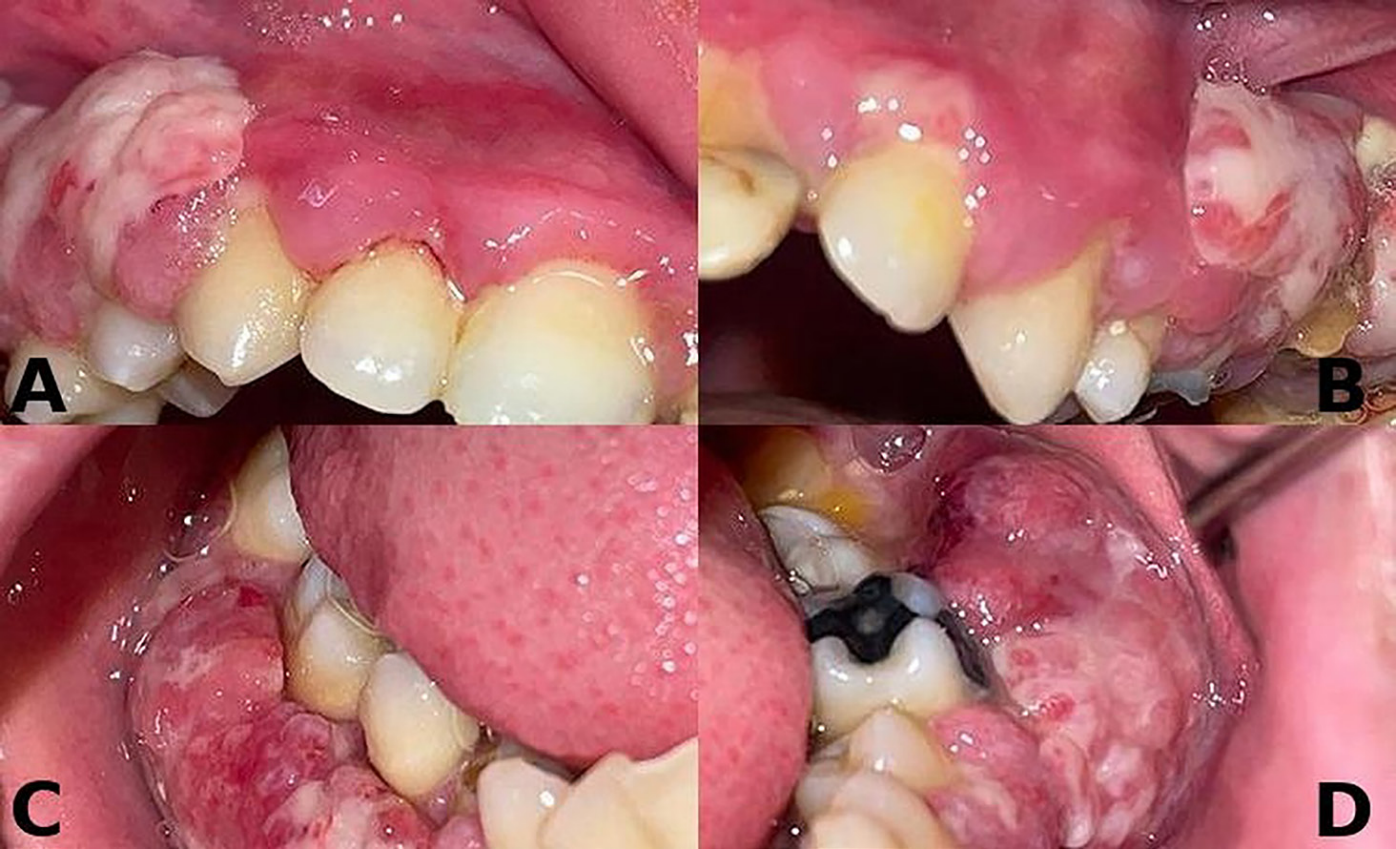
A,B,C,D: Intraoral view of the patient showing abnormal hyperplastic lesions with pedunculated appearance on the buccal surfaces of all four quadrants, separable with the help of a periodontal probe.

**Figure 2:**
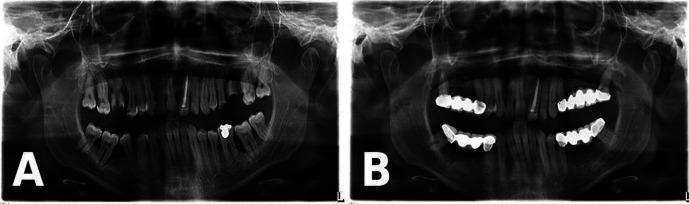
A: Panoramic radiograph demonstrating advanced vertical and horizontal alveolar bone loss with severe furcation involvement, particularly around the posterior molar teeth. B: Panoramic radiograph demonstrating stable bone levels around the remaining teeth with no progression of bone loss after two years of follow-up, along with completed fixed prosthetic rehabilitation.

**Figure 3:**
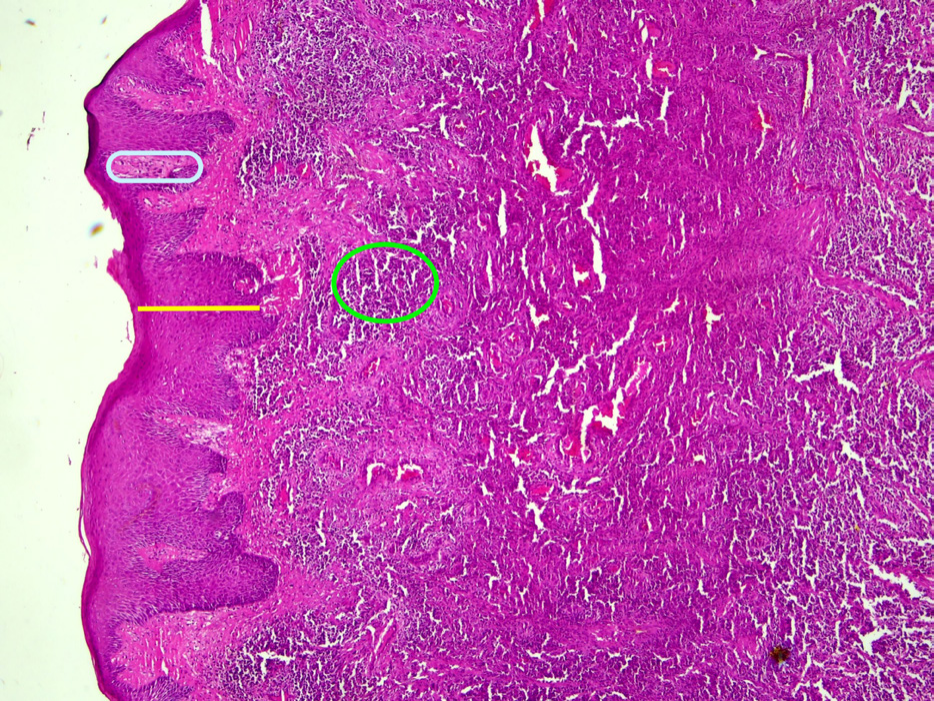
Histopathological image demonstrating epithelial hyperplasia (yellow area), widespread subepithelial fibrin accumulation (blue area), and mixed inflammatory infiltrate composed of histiocytes and lymphocytes (green area), consistent with ligneous periodontitis.

A 23-year-old male presented to the Ondokuz Mayis University Faculty of Dentistry, Department of Oral and Maxillofacial Radiology for evaluation of gingival enlargement with bleeding in 2022. His medical history included hydrocephalic birth, delayed exfoliation of primary teeth, late eruption of permanent teeth, and recurrent gum problems. Intraoral examination revealed widespread gingival hyperplasia and erythema, with prominent white-red lesions observed in all quadrants, particularly in the posterior regions (Fig. 1). Clinically, these lesions were pedunculated and could be separated using a periodontal probe, causing hypersensitivity in the affected areas. Panoramic radiography showed extensive bone loss and severe furcation defects in both the maxilla and mandible, particularly affecting the posterior teeth (Fig. 2A). Based on the clinical and radiographic findings, a plasminogen blood test was requested, which revealed a markedly reduced plasminogen level of 27% (reference range, 70–130%), supporting the preliminary diagnosis of LP. Phase I periodontal treatment was performed. This included supragingival plaque and calculus removal, oral hygiene instruction, and subgingival root planing with soft tissue curettage under local anesthesia. Prior to surgical procedures, hematology consultation recommended the administration of two units of fresh frozen plasma (FFP). In collaboration with the oral and maxillofacial surgery department, teeth with hopeless prognosis were extracted and extensive soft tissue enlargements in the posterior molar regions were excised in staged surgical sessions. The biopsy specimen taken during the first surgical session was sent for histopathological examination. Following each surgical session, the patient was prescribed antibiotics (amoxicillin + clavulanic acid 1 g, twice daily for 5 days), analgesics (dexketoprofen 25 mg, twice daily for 5 days), and a chlorhexidine mouthwash (0.12%, three times daily for 5 days). The histopathological examination of the biopsy specimen revealed focal epithelial erosion, hyperplasia, parakeratosis, and subepithelial homogeneous eosinophilic material accumulation. Additionally, chronic active inflammation and vascular proliferation were observed in the lamina propria, findings consistent with LP (Fig. 3). The patient was subsequently referred to the Department of Medical Genetics for further evaluation. During this period, periodontal maintenance phases were regularly performed, and no recurrences were observed. Sequencing of the PLG gene (NM_000301) revealed a novel c.2087G>C (p.Arg696Pro) variant along with a previously reported c.147T>A (p.Cys49Ter) variant. The detected variants were confirmed by Sanger sequencing, which also demonstrated co-segregation within the family. Following completion of surgical and periodontal treatments, and after a two-year follow-up period without recurrence, the patient’s missing teeth were restored with fixed prosthetic rehabilitation (Fig. 2B). The patient provided written informed consent for the publication of all clinical data and images presented in this case report.

## Discussion

Ligneous conjunctivitis (LC) was first described by Bouisson in 1847 (4). Approximately 150 years later, oral lesions associated with conjunctivitis were identified and described in detail (5,6). Gunhan *et al*. first described LP as a rapidly progressing and destructive periodontal condition characterized by severe alveolar bone loss and lobulated gingival overgrowths, underlining the aggressive course of this disorder (2). LC is recognized as the most common and classical manifestation of type 1 PLG deficiency, reported in approximately 80% of cases, with a noted female predominance (ratio ranging from 1.4:1 to 2:1) (7). Some cases have also been associated with congenital occlusive hydrocephalus (8,9). In our patient, congenital hydrocephalus and severe oral findings were present, but notably without ocular involvement. Based on the clinical oral findings, a preliminary diagnosis of ligneous periodontitis was established, and a plasminogen blood test was requested. The histopathological analysis of the biopsy specimen confirmed the diagnosis. In the differential diagnosis, medication-related gingival enlargement, leukemic infiltrations, necrotizing ulcerative gingivitis, and desquamative gingivitis caused by systemic and local factors should be considered (10,11). Therefore, histopathological examination combined with hematologic evaluation is essential to avoid misdiagnosing LP as other clinically similar conditions. This underscores the importance of dentists in recognizing early signs of type 1 PLG deficiency, as oral findings may represent the initial or even the sole clinical manifestation. Careful oral examination can therefore play a pivotal role in the early detection of systemic diseases.

Once a preliminary diagnosis is established and differential diagnoses are excluded, understanding the nature and prognosis of the condition is essential. Gingival lesions associated with LP are known to be progressive and typically result in tooth loss. Despite various surgical and periodontal treatment attempts documented in the literature, most interventions have been unsuccessful in achieving long-term resolution of these lesions (12-14). In our case, performing an early excisional biopsy was essential for making the correct diagnosis and planning the appropriate multidisciplinary treatment. Thorough phase 1 periodontal therapy, effective oral hygiene motivation, and FFP replacement prior to surgical procedures were key factors in the successful management of this case. Notably, no recurrence of lesions, no progression of alveolar bone loss, and no worsening of other mucosal sites were observed during the two-year follow-up period.

## Conclusions

This case report highlights the importance of early diagnosis and multidisciplinary management in patients with ligneous periodontitis, a rare but severe manifestation of plasminogen deficiency. Early periodontal intervention, combined with appropriate surgical treatment, hematologic support, and regular follow-up, can lead to successful long-term outcomes without recurrence. Furthermore, advancements in genetic studies and the potential use of recombinant plasminogen replacement therapy may open new doors for more targeted and definitive treatment strategies.

## Data Availability

The datasets used and/or analyzed during the current study are available from the corresponding author.
